# Promoting mental health in nursing students during clinical training: a phenomenological analysis of a psychoeducational intervention

**DOI:** 10.1080/17482631.2026.2637800

**Published:** 2026-02-28

**Authors:** Pilar González-Sanz, Clara Isabel Posada-Abadía, Oscar Pajares González-Gallego, Juan Luis González-Pascual, Beatriz Jiménez-Gomez, Pedro Belinchón-De Miguel

**Affiliations:** a Universidad Europea de Madrid, Department of Nursing, Faculty of Medicine, Health and Sports, Madrid, Spain

**Keywords:** Clinical practice, mental health care, psychoeducational program, psychological stress, resilience, students nursing, phenomenology, qualitative research

## Abstract

**Introduction:**

Mental health issues among young people are increasingly concerning and affect nursing students. The academic environment is recognised as a source of stress, compounded by the demands of clinical settings during practical training. During clinical placements, students face emotionally challenging situations that may compromise their mental health and academic performance. It is the responsibility of nurse educators to implement strategies that help students develop the necessary skills for managing stress and anxiety, as well as effective coping and communication. A psychoeducational programme designed for this purpose is presented.

**Aim:**

To explore the experiences of nursing students in participating in a psychoeducational program and its potential influence on the promotion of their mental health.

**Method:**

A qualitative phenomenological study was conducted using semi-structured interviews with 11 nursing students who completed the psychoeducational programme. Data were analysed through thematic coding and phenomenological reduction.

**Discussion:**

Key emerging superordinate themes included: My safe space: the group as support and help network; Seeing things differently: reflection and change; Confidence, safety, and self-esteem; Improvement in interpersonal relationships. The psychoeducational programme provided spaces for reflection and emotional support, contributing to a healthier clinical experience. The findings are consistent with previous studies highlighting the importance of early mental health interventions for students.

**Conclusion:**

Implications for practice. Integrating psychoeducational programmes into clinical training may enhance emotional wellbeing and promote mental health among students.

## Introduction

Depression, anxiety, and behavioural disorders are among the leading causes of illness and disability in young people and adolescents worldwide (World Health Organisation, 2024). Nursing students, who typically fall within this age group, are particularly vulnerable to mental health issues such as anxiety, stress, and depression during their academic journey or in later years (Karaca et al., 2019; Mohamed et al., 2025; Stubin & Dahan, 2024). The highest prevalence of depression—up to 41%—is found among younger students aged 17 to 28 (Tung et al., 2018), who also exhibit greater cognitive distortions associated with stress and anxiety (Alwawi & Alsaqqa, 2023). Moreover, this university stage is often when substance use begins, which may lead to further mental health disorders (Liu et al., 2019).

In nursing education, the university environment is perceived as a significant source of stress, requiring a delicate balance between academic and clinical demands (Enns et al., 2018; Terp et al., 2019). The clinical setting presents a real challenge for students, who must navigate complex, uncertain, and high-responsibility situations. These experiences often lead to feelings of insecurity and inefficacy, especially in interpersonal interactions with patients, families, and healthcare professionals (Hussien et al., 2020; Z. S. Li & Hasson, 2020; Simpson & Sawatzky, 2020; Stubin, 2020). Clinical placements are formative experiences that shape students’ professional identity and development, significantly influencing their mental wellbeing (Aryuwat et al., 2024; Thomas & Asselin, 2018).

In light of this reality, it is therefore the responsibility of nurse educators to implement appropriate measures to promote students’ mental wellbeing (Stubin & Dahan, 2024).

This mental wellbeing, an essential component of overall wellbeing, is linked to the strengthening of protective factors and to the empowerment of students and the wider educational community to respond healthily to academic and clinical challenges. This perspective aligns with the World Health Organisation’s (2025) conceptualisation of mental health promotion, which focuses on enhancing psychological, emotional and social resources that support full development (World Health Organisation, 2025).

The scientific literature shows that improvements in mental wellbeing are associated with reductions in stress and anxiety through various interventions—such as positive psychology, cognitive-behavioural therapy, mindfulness, meditation, music therapy, aromatherapy and laughter therapy—implemented in face-to-face, online, hybrid formats or via mobile applications (Aksu & Ayar, 2023; Baldassarini et al., 2022; Li et al., 2018; Russell et al., 2025; Wang et al., 2022). Other studies highlight the importance of support systems—social, peer-based or institutional— as key elements for competent supervision and effective collaboration between teachers and students, contributing to the development of clinical competence (Devi et al., 2021; Edwards et al.,^2022^; Ferreira et al., 2021). Likewise, interventions focused on stress management, emotional regulation and assertive communication appear to support the development of resilience and self-efficacy, strengthening good mental health, an area in which further research is needed (Visier-Alfonso et al., 2024; Martínez et al., 2025; Yosep et al., 2025).

With this purpose, an innovative psychoeducational programme was designed and implemented, led by a mental health and a clinical learning educator, to support students in developing essential skills in stress and anxiety management, assertive communication, reflection and self-awareness, among others, enabling them to approach their clinical placements in a calm, positive and healthy manner.

The programme, delivered in a group format, consisted of eight sessions, held once a week over two months, each lasting 90 minutes. It sought to create an environment of respect and trust that fostered dialogue, confidence and peer learning, addressing students’ concerns, fears and needs while integrating topics (outlined in Table I) and techniques aimed at developing essential emotional and communication skills. Group-based activities were employed, using guided questioning to facilitate debate, active listening and reflection; modelling by the facilitator to support the development of assertive communication, problem analysis and problem-solving; and the use of active coping strategies and relaxation techniques to promote emotional self-regulation.

**Table I.** Psychoeducational programme: session planning and content.

Phase	Content	Duration
Opening	Sharing of daily clinical placement experiences	15 minutes
Development	Psychoeducation—participatory lecture format: Session 1: Emotional intelligence Session 2: Challenging situations in clinical placements. Problem-solving Session 3: Key strategies for emotional regulation Session 4: Communication styles. Assertiveness Session 5: Stress and anxiety Session 6: Stress and anxiety II Session 7: Self-concept and self-esteem Session 8: Resilience and self-based communication	30 minutes
Reflection	Reflection and group discussion on the resources covered and lived experiences during placements	30 minutes
Closing	Session wrap-up: brief word or phrase summarising what was learned	15 minutes

The aim of the present study was to explore how nursing students experienced their participation in the programme and how this experience may relate to the potential.

## Methodology

### Design

This research has been designed and written following the COREQ (Consolidated Criteria for Reporting Qualitative Research) checklist, which aims to ensure transparency, rigour, and completeness in the reporting of qualitative studies. The study was framed within an interpretative phenomenological perspective, aimed at gaining an in-depth understanding of the lived experiences of nursing students during their participation in a psychoeducational programme, as well as the meanings constructed to give sense to that experience. This approach allowed for a structured examination of the lived experience associated with participation in the psychoeducational programme, guiding its analysis and interpretation in accordance with the essence of subjective experience (Van Manen, 2014).

Attention was focused on the uniqueness of each narrative, which allowed for greater expressive richness through individual interviews. Throughout the process, the researchers maintained an attitude of openness, sensitivity and respect towards emerging meanings, avoiding the imposition of external interpretations. The research process was conducted under a reflexive-relational orientation, with a strong practical emphasis, in line with the principles proposed by Finlay (2016) and Smith et al. (2021). An interpretative phenomenological analysis was conducted following the approach proposed by Smith et al. (2009).

### Contextualisation

In Spain, within the framework of the Bologna Process, nursing students undertake a total of 2,300 hours of external academic placements, equivalent to 90 ECTS credits, distributed across the four years of their undergraduate training (Orden CIN/2134/2008, de 3 de Julio, 2008).

Students rotate through various healthcare centres and units, supervised by a clinical nurse and a clinical learning educator (nurse employed as university faculty), who oversees the achievement of the learning outcomes defined in the curriculum. Their adaptation and progress within the clinical placement setting are continuously monitored. The clinical nurse is present daily with the students, providing ongoing support and guidance in their practical activities. In addition, the clinical learning educator conducts weekly visits to meet with the students, identify any issues, monitor their learning progress, and foster reflective learning. An intermediate evaluation is carried out by the clinical nurse and reviewed jointly with the students and the clinical learning educator, enabling the implementation of improvement plans prior to the final evaluation.

The implemented psychoeducational programme was a pilot project for second-year nursing students at the beginning of the academic year when their clinical practice started (November and December 2023).

### Participants

**Figure 1.**
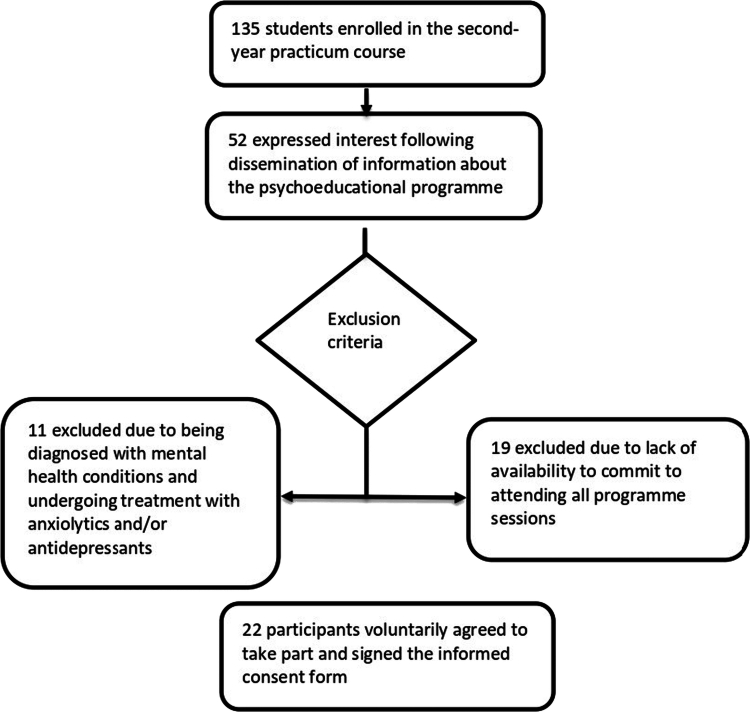
Participant selection process for the psychoeducational programme.

The programme was introduced to a total of 135 second-year nursing students enroled in clinical placement modules. Following the inclusion and exclusion criteria, from 52 interested participants, 11 were excluded due to previous mental health issues and 19 due to lack of availability, yielding a final sample of 22 students (Figure 1). They were divided into two groups—morning and afternoon—according to their assigned clinical placement shifts at the hospital. The students attended all the programme sessions in the same group.

Participants were selected through purposive sampling from the 22 students who participated in the programme, based on their engagement, interest, and capacity for verbal and reflective expression. To enhance the breadth of perspectives, we also applied a maximum-variation strategy, ensuring heterogeneity in age, sex, concurrent employment in healthcare.

Data saturation was reached with 11 participants (Creswell & Poth, 2018; Moser & Korstjens, 2018). Among the participants, 82% were female and the average age was 24 years. None reported having dependents under their care, and 64% combined their studies with employment in the healthcare sector.

### Data collection

Data were collected between May and September 2023 through semi-structured interviews guided by open-ended questions. The interview guide was developed based on the contents of the psychoeducational programme and its application to clinical placement situations or other everyday contexts (Moser & Korstjens, 2018) (Table II).

**Table II.** Individual interview guide.

Themes	Questions
Emotional Management	What positive and negative emotions can you identify?
	During your clinical placement, which emotions did you experience?
	How does identifying them help you?How do you think this programme has helped you in this regard?
Stress and Anxiety Management	How do you usually manage stress and anxiety, and what triggers them?
	During your placement, what situations caused stress or anxiety, and how did you cope?
	How would you handle similar situations next time? What has this programme taught you about it?
Self-awareness and Self-esteem	How do you perceive yourself?
	What are your strengths? How did you perceive yourself during your placement?
	How has the programme helped you in this aspect?
Purpose and Motivation	What are your goals during clinical placements and in your nursing studies overall? How motivated do you feel?
	What made you feel proud during your daily practice? What would you like to achieve in the short term?
	What motivated you to participate in this programme? How has it helped you in this regard?
Effective Communication	How did you feel when interacting with patients, families, and the professional team?
	In what situation did you practise speaking from the “I” perspective?
	What did this bring you?
	How has the programme helped you in your relationships and communication style?
	What do you consider most important in communication?
Autonomy and Resilience	How do you respond to uncertainty? How do you react to unexpected situations?
	How did you adapt to the clinical unit? How did you manage unforeseen situations during your placement?
	Describe your usual support network and the one you had during clinical placements. When do you seek help from your support network? How do you feel when doing so?
	How did the programme help you in this regard?

The interviews were conducted by three members of the research team (JLG, BJ, PB), all of whom were nurses with a doctoral degree and had experience in qualitative research, who had not participated in the programme sessions. Interviews were scheduled according to participants’ availability and took place either in university meeting rooms or via online platforms when in-person meetings were not feasible (Archibald et al., 2019; Tailor et al., 2024). Each interview lasted approximately 60 minutes and was fully transcribed by the respective researcher.

## Ethical considerations

The study was approved by the Research Ethics Committee of Hospital Universitario de Getafe (reference CEIm24/62), to which it was referred by the Research Commission of the collaborating university for ethical review, within the framework of an existing institutional agreement. None of the authors have any employment or contractual relationship with Hospital. The study adhered to the principles outlined in the Declaration of Helsinki and complied with current legal regulations on biomedical research (Law 14/2007, of June).

All participants were informed prior to providing voluntary consent about the purpose of the study, the nature of their participation, potential risks and benefits, and the procedures for handling personal data. All participants gave written consent prior to their inclusion in the study. Confidentiality was ensured in accordance with the provisions of Organic Law 3/2018 of 5 December on the Protection of Personal Data and the Guarantee of Digital Rights.

## Data analysis

An interpretative phenomenological analysis was conducted following the approach proposed by Smith et al. (2009), by two researchers (PG, CP), both nurses with a doctoral degree and had expertise in qualitative research.

The analysis followed six iterative stages (Smith et al., 2009; 2021). First, both researchers independently read each transcript several times to achieve immersion and develop an initial understanding of participants’ experiences. Second, detailed notes were recorded to capture content and nuances of expression. Third, these notes were synthesised into emergent subthemes grounded in the data. Fourth, subthemes were clustered within each case to identify patterns and relationships, followed by a researcher meeting for triangulation. Fifth, the process was repeated for each interview, bracketing previous subthemes to maintain an idiographic focus, with four additional meetings to compare analyses. Finally, patterns across cases were examined to develop superordinate themes, supported by transcript evidence, and refined through three researchers discussions.

An overview of the subthemes and superordinate themes is presented in Table III.

**Table III.** Superordinate themes and subordinate themes revealed.

Superordinate themes	Subordinate themes
My safe space: the group as support and network	Negative emotions during placements: fear, helplessness, loneliness, and low self-esteem
	No fear of being judged
	Sharing experiences and listening to each other
	Identification and expression of emotions
Seeing things differently: reflection and motivation for change	Change in perception of problematic situations
	Pause, reflect, and become aware
	Not everything is always positive: finding balance
	Other points of view
Confidence, security, and self-esteem	Greater confidence in myself
	Able to face the problem: timely, calmly, and with the right person
	The problem is not mine
	Learning and continuing to grow
Improvement in interpersonal relationships	Reflection and self-criticism before judging others
	Putting oneself in the other’s place; empathy
	Listening to others
	Listening from patients
	Helping others understand how we feel

## Positionality and trustworthiness

The research team comprised nursing academics and clinical professionals with complementary expertise that enriched the qualitative study.

PG (female, nurse, PhD) served as Clinical Learning Coordinator for the undergraduate nursing programme.

CP (female, nurse, PhD) conducted qualitative research with vulnerable populations.

OP (male, Specialist Mental Health Nurse, MSc) contributed expertise in mental health practice and education.

JLG (male, nurse, PhD) coordinated the undergraduate nursing programme.

BJ (female, nurse, PhD) served as clinical learning educator in the undergraduate nursing programme.

PB (male, nurse, PhD) also served as clinical learning educator in the undergraduate nursing programme.

Trustworthiness was ensured through a phenomenological design aligned with the research question, together with consistent sampling and data collection strategies. Reflexivity was practiced throughout the research process to minimise researcher influence, and an inductive analytical approach avoided preconceived assumptions while honouring participants lived experiences. Reliability was strengthened through team triangulation and iterative analytical discussions.

### Findings

The analysis of the interviews yielded four superordinate themes (Table III), which provide insight into the participants’ experiences with the psychoeducational programme.

### Superordinate theme 1. My safe space: the group as support and a network of help

This superordinate theme is composed of the cluster of four subthemes: Negative emotions during placements—fear, helplessness, loneliness, and low self-esteem; No fear of being judged; Sharing experiences and listening to each other; and Identification and expression of emotions.

The students began their clinical placements and attended the university once a week to take part in the programme. Although not all of them knew each other beforehand, a meaningful space and time for trust, mutual understanding, and support gradually emerged. This weekly session offered an opportunity to express themselves and their emotions freely and share their experiences with peers without feeling judged or assessed. The clinical environment can often be hostile, generating discomfort and a need for emotional release.

P2. *“… we created a good atmosphere, there were only a few of us, and we built a space of trust, so everyone shared their stuff without… without fear of backlash or being judged or anything…”*

P4. *“Sometimes I spend the whole week in a place I don’t like or where I’ve been treated badly, and just coming here for these three hours helps to balance things out a bit, because in the end I can let it all out, I know I’m understood, I’m listened to and… it helps me cope with what’s going on… it’s a big help for all of us.”*

Not feeling judged or evaluated enabled them to communicate more freely and to develop their ability to listen to one another. Gaining alternative perspectives and sharing concerns—some of them shared—helped them feel less alone and recognise each other’s experiences. This peer-to-peer learning validated their ideas and fostered a sense of trust, identity, and belonging.

P7. *“Being able to share a space with people who are going through the same things as me… you feel like you’re not alone, that you’re being heard, that you have a space where you can talk about how you feel, what’s worrying you… I think having this support network is essential…”*

P3. *“…There you see and hear other classmates’ points of view, and the teacher’s too… and it helps, because I have my own way of being and dealing with situations, but then you see that someone else would handle it differently… it’s not better or worse, just another way that might work…”*

#### Superordinate theme 2. Seeing things differently: reflection and change

This superordinate theme is composed of the cluster of four subthemes: Change in perception of problematic situations; Pause, reflect, and become aware; Not everything is always positive: finding balance; and other points of view.

During the sessions, students shared the difficulties they were facing, and when these involved interpersonal relationships, they were encouraged to “put themselves in the other person’s position”. This meant imagining themselves in the place of the supervisor who had corrected them in front of a patient, the one who had spoken to them harshly, or the one who failed to greet them or simply ignored them. Through this process (pause, reflect, become aware) the students came to understand the importance of empathy and recognised that they were not responsible for other people’s behaviour. They began to realise that when they were treated poorly, the issue did not lie with them.

P2. *“It’s helped me to take things more calmly, not take it personally or get so upset… and to go in with a different attitude… because you always have to adapt…”*

P10. *“If there’s a problem, you must face it, think it through, talk about it and see what you’re doing wrong. And why… sometimes the way people treat us, which we think are attacks, aren’t really… and it’s us who are doing something wrong…”*

In this way, they also came to understand that certain aspects lay beyond their control and that they would therefore always need to adapt. They recognised that reality does not always align with expectations and that this mismatch can generate frustration; not everything will unfold as one hope. They were able to identify both positive and negative elements of their clinical placements, seeking a constructive balance in their experiences and, in doing so, strengthening their resilience.

P11. *“…I’m more aware of all the things that can happen, both good and bad, and that you must deal with them in the best way possible. If there’s a bad moment, well, there’ll be another one that balances it out…”*

### Superordinate theme 3. Confidence, security, and self-esteem

This superordinate theme is composed of the cluster of four subthemes: Greater confidence in myself; Able to face the problem: timely, calmly, and with the right person; The problem is not mine; and Learning and continuing to grow.

The sessions also addressed the identification and management of emotions, encouraging students not to fear speaking up or expressing their feelings when faced with a conflict. They were guided to “speak from the self”, without offending or pointing at others. This approach gave them a sense of confidence, freeing them from the fear of potential repercussions or the anticipatory worries they previously experienced, including concerns about failing a placement due to their supervisor’s judgement.

P9. *“…I was able to speak up and say I didn’t understand, to my tutor, in a respectful way… and if I hadn’t been part of the group, I wouldn’t have done it… it gave me a different way of thinking… I mean, it was about facing the situation, but in a constructive way.”*

Moreover, as they gradually realised that they were capable of approaching challenging situations differently and felt equipped with new strategies, they began to transfer these skills to other areas of their lives, both academically and personally. They reported greater confidence, a stronger sense of security, and an overall improvement in their self-esteem.

P8. *“…I’ve grown a lot by doing this… it’s helped me have more control over everyday things… even if sometimes things slip through… it’s helped me to be more in control and calm when doing things… I feel like I’m not the same person I was last year.”*

P3. *“…I’ve gained more confidence in myself, and I think I’ve grown overall thanks to being in this programme… I’ve used a lot of tools, especially in the OSCE and performance evaluations… honestly, it’s helped me and made those assessments easier.”*

P4. *“…now I feel capable of doing things I wouldn’t have done before… for example… I wouldn’t be here today… I’m shy… my self-esteem has gone up.”*

### Superordinate theme 4. Improvement in interpersonal relationships

This superordinate theme is composed of the cluster of five subthemes: Reflection and self-criticism before judging others; Putting oneself in the other’s place: empathy; Listening to others; Listening from patients; and Helping others understand how we feel.

The increase in confidence and sense of security led to a notable improvement in communication, and consequently, in interpersonal relationships. Students became more aware of the importance of effective communication. Effective communication grounded in empathy and active listening became a conscious process and appeared to be put into practice.

P8. *“Yes, communicating in a more assertive way and changing how I speak… reformulating my words… even with my parents… it’s helped me do things better… they’ve noticed it too.”*

They gradually acquired tools that enabled them to build more effective relationships with patients and their families. Although they had previously encountered these aspects in a theoretical manner and were aware of their importance, it was through their shared group experience that they came to understand and internalise them in a different, more meaningful way. From active listening, as previously discussed, to the importance of attentive observation of patients and their families in relation to non‑verbal communication, these are key aspects for achieving effective communication.

P10. *“With patients I’ve learned to be more empathetic. You can’t always communicate well with them because they’re not in a good state… so I pay more attention to non-verbal language and try to be careful and understand them… in the end, you connect…”*

P11. *“This programme has mainly helped me with active listening… how you must listen to everything, pay attention… everything that’s happening in the hospital. If there’s a problem, you must listen to both sides… listen to yourself, how you’re feeling, and put yourself in the other person’s shoes and understand how they’re doing… I’ve already brought this into my daily life… not just in clinical placements, but also in my personal life.”*

## Discussion

This study has helped to elucidate how a group-based educational programme, led by a nurse educator and mental health specialist, implemented in parallel with the clinical practice, with a focus on emotional management, stress, and anxiety, influenced the practical experience of nursing students.

### My safe place: the group as support and a network of help

Our study confirms the importance of an effective support system that promotes the emotional wellbeing of students within both university and clinical settings (Albaqawi et al., 2025), helping them to manage and alleviate the stressors they perceive and which may undermine their motivation to learn (Zhang et al., 2020) or even lead them to consider withdrawing from their studies (Sharpnack, 2024; De Alencar et al., 2025). This group-based support served as a form of social connection, fostering in students a shared sense of meaning, purpose, and collective efficacy that contributes to good mental health, as highlighted in previous research (Haslam et al., 2022; McIntyre et al., 2018). Moreover, peer listening and understanding not only provided a sense of mental wellbeing (Kachaturoff et al., 2020) but also facilitated peer learning (Cuesta Martínezc et al., 2024; Jacobsen et al., 2022), reducing the stress they experienced in their interactions with faculty and allowing them to engage without feeling judged or evaluated (Bhurtun et al., 2019; Stubin, 2020).

### Seeing things differently: reflection and change

Students reported a shift in their perspective and coping strategies, incorporating reflection before acting or making decisions. This process led to increased self-awareness and self-regulation, enhancing their personal resources and flexibility (Ching et al., 2020). As observed in the study by Froneman et al., (2023), critical self-enquiry and reflection on their experiences enabled students to derive new meanings and perspectives.

Such emotional regulation and reflective practice contributed to the development of empathy, understanding, and other essential skills for clinical practice (Salem et al., 2025). Reflection was also recognised as an internal protective factor that may reduce stress and foster resilience among nursing students (Bui et al., 2023; Slimmen et al., 2022).

Furthermore, our findings support previous evidence that resilience and low stress levels are predictors of improved mental wellbeing (Li & Hasson, 2020).

### Confidence, security, and self-esteem

Our findings confirm that emotional regulation and reflective practice contributed to increased confidence, a greater sense of security, and enhanced self-esteem among students. There was a clear transformation in their self-perception enabled them to approach situations previously regarded as problematic in a more constructive manner.

Confidence emerged as a key element in participants’ personal development. Their ability to speak openly, share doubts, and face situations without fear represented a significant shift (McVeigh et al., 2021a; McVeigh et al., 2021b).

This sense of security was further strengthened by students’ acceptance of others’ perspectives, their ability to acknowledge negative emotions, and their understanding of emotional diversity within clinical environments. Emotional competencies were thus developed (Froneman et al., 2023; Mlinar Reljić et al., 2019) and were directly linked to students’ self-concept and the enhancement of their self-esteem (Dancot et al., 2023; Lundell Rudberg et al., 2022).

Our study supports the importance of accompaniment as a key factor in students’ development (Dancot et al., 2024).

This combination of emotional, reflective, and relational experiences enabled students to recognise themselves as active agents in their own learning process, with greater motivation and resources to engage in it (Lavoie-Tremblay et al., 2022; Manti et al., 2022; Oostvogels et al., 2018).

### Improvement in interpersonal relationships

The increase in personal security, confidence, and self-esteem led to a notable improvement in the quality of students’ interpersonal relationships. The development of empathy, the ability to express emotions from the self and active listening emerged as key tools for building respectful, meaningful, and healthy connections in professional, personal, and family contexts (Swan, 2021; Xie & Derakhshan, 2021). Emotional regulation enabled them to establish safer and more constructive interpersonal relationships (Elsayes & Abo-Elyzeed, 2021; Lee & Park, 2022).

Participants became more aware of the importance of non-verbal communication and showed greater willingness to understand others in vulnerable situations, fostering more collaborative relationships and improved interpersonal communication (Cid Gutiérrez & Urrutia Martinez 2022; Mägi et al., 2024)This enhanced understanding of emotional and social diversity within clinical environments promoted empathy, respect, and effective communication (Antón-Solanas et al., 2021). Furthermore, the practice of active listening and attention to others’ emotions and needs extended into their family lives (Manusov et al., 2020; Petersen, 2019) contributing to greater mental wellbeing.

Given that poor interpersonal relationships can increase students’ vulnerability to negative emotions and the development of depression (Yan et al., 2025), our findings support the opposite effect: improved mental wellbeing when interpersonal relationships are strengthened—particularly relevant in educational and clinical contexts (Huang et al., 2020).

Therefore, the integration of psychoeducational programmes during clinical placements could be considered as part of the nursing curriculum, contributing to improved interpersonal relationships and, ultimately, to the quality of care provided (Ng, 2020).

## Strengths and limitations

The study was planned and reported following the COREQ checklist, which ensures clarity, transparency and facilitates reproducibility.

This study is not without limitations. It was conducted within a specific curricular and institutional context, involving second-year undergraduate nursing students whose learning trajectories were shaped by the structure of their degree programme. In addition to undertaking the psychoeducational programme while simultaneously completing their clinical placements on their second year, these students had already completed a four-week hospital rotation during their first year, which may have influenced both their perceptions and the way they engaged with the intervention.

Additionally, the study was carried out within a single university and a specific geographical context, which may limit the generalisability of the findings.

## Conclusions

The psychoeducational programme enabled students to feel safe and confident in expressing their fears, doubts, and opinions without pressure. Their responses, accepted and validated by peers, contributed to the development of a sense of belonging, purpose, and collective efficacy—factors known to support mental health.

Through listening, critical reflection, and self-inquiry, students improved their emotional regulation and mental wellbeing, reducing anxiety and stress while increasing flexibility and resilience.

This shift in self-perception also enhanced active listening and other skills essential for effective communication, leading to significant improvements in interpersonal relationships and, consequently, in self-esteem.

We conclude that programmes of this nature are not only feasible and necessary within higher nursing education, but also effective in supporting students’ emotional development and mental wellbeing.

## Relevance for clinical practice

The findings support the implementation of psychoeducational programmes by nurse educators that promote mental health and emotional wellbeing from the beginning of undergraduate training, particularly during clinical placements. Holistic support for students—focused on both personal and professional development—may help prevent academic and professional dropout.

Emotional management, self-awareness, the enhancement of coping processes, and communication skills are essential tools that students need to develop in order to achieve appropriate personal and professional growth, as well as to strengthen their overall nursing training.

Furthermore, learning self-care strategies can enhance students’ awareness and facilitate the transfer of skills needed to promote mental health in their future clinical practice.

## Declaration

The authors state that they have not received financial support and have no relationships that could be considered a conflict of interest in relation to this work.

## Disclaimers

The interpretations and reflections presented in this article are solely those of the authors and do not necessarily represent the official position of their institution. No potential conflict of interest was reported by the author(s).

## Data Availability

Research data supporting this publication are available upon reasonable request to the corresponding author, subject to ethical and privacy considerations.
